# Quercetin as a Therapeutic Product: Evaluation of Its Pharmacological Action and Clinical Applications—A Review

**DOI:** 10.3390/ph16111631

**Published:** 2023-11-20

**Authors:** Mohd Aamir Mirza, Syed Mahmood, Ayah Rebhi Hilles, Abuzer Ali, Mohammed Zaafar Khan, Syed Amir Azam Zaidi, Zeenat Iqbal, Yi Ge

**Affiliations:** 1 Department of Pharmaceutics, School of Pharmaceutical Education and Research, Jamia Hamdard, New Delhi 110062, India; aamir.jhu07@gmail.com (M.A.M.); amirazamzaidi1@gmail.com (S.A.A.Z.); 2 Department of Pharmaceutical Technology, Faculty of Pharmacy, Universiti Malaya, Kuala Lumpur 50603, Malaysia; 3 INHART, International Islamic University Malaysia, Jalan Gombak, Kuala Lumpur 53100, Malaysia; ayah.hilles90@gmail.com; 4 Department of Pharmacognosy, College of Pharmacy, Taif University, Taif 21944, Saudi Arabia; abuali@tu.edu.sa; 5 School of Pharmacy, Queen’s University Belfast, Belfast BT9 7BL, UK

**Keywords:** quercetin, flavonoids, therapeutic profile, nanoformulation, lipid nanoparticles

## Abstract

Quercetin is the major polyphenolic flavonoid that belongs to the class called flavanols. It is found in many foods, such as green tea, cranberry, apple, onions, asparagus, radish leaves, buckwheat, blueberry, broccoli, and coriander. It occurs in many different forms, but the most abundant quercetin derivatives are glycosides and ethers, namely, Quercetin 3-*O*-glycoside, Quercetin 3-sulfate, Quercetin 3-glucuronide, and Quercetin 3′-metylether. Quercetin has antioxidant, anti-inflammatory, cardioprotective, antiviral, and antibacterial effects. It is found to be beneficial against cardiovascular diseases, cancer, diabetes, neuro-degenerative diseases, allergy asthma, peptic ulcers, osteoporosis, arthritis, and eye disorders. In pre-clinical and clinical investigations, its impacts on various signaling pathways and molecular targets have demonstrated favorable benefits for the activities mentioned above, and some global clinical trials have been conducted to validate its therapeutic profile. It is also utilized as a nutraceutical due to its pharmacological properties. Although quercetin has several pharmacological benefits, its clinical use is restricted due to its poor water solubility, substantial first-pass metabolism, and consequent low bioavailability. To circumvent this limited bioavailability, a quercetin-based nanoformulation has been considered in recent times as it manifests increased quercetin uptake by the epithelial system and enhances the delivery of quercetin to the target site. This review mainly focuses on pharmacological action, clinical trials, patents, marketed products, and approaches to improving the bioavailability of quercetin with the use of a nanoformulation.

## 1. Introduction

**Figure 1:**
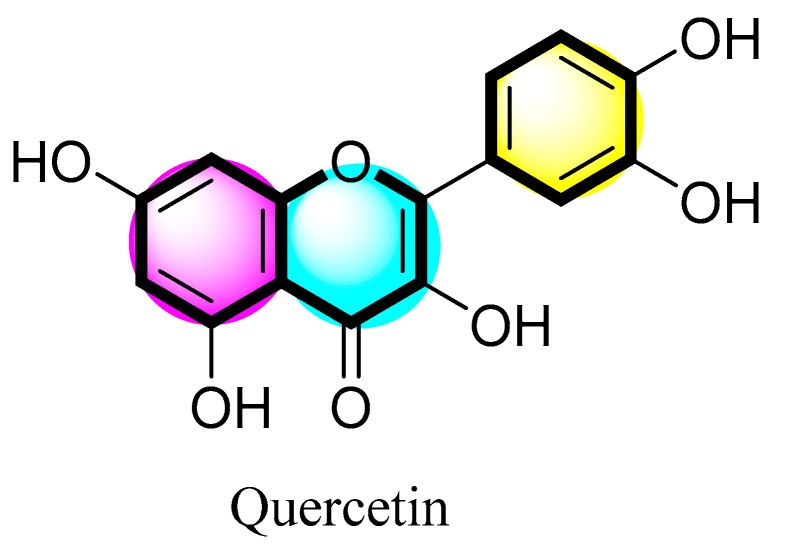
Molecular structure of quercetin.

In nature, flavonoids are ubiquitous in the form of benzo-γ- pyrone derivatives. They are mostly found in vegetables, flowers, and plants. Flavonoids have different structural forms with intriguing biological features and can play a significant function in the immune system of the body. Multiple studies have revealed the positive effects of flavonoid-rich diets. There are about 4000 different forms of flavonoids in nature, including subclasses such as flavones, isoflavones, flavanones, and chalcones. Flavonoids exhibit key biological actions such as antioxidant, hepatoprotective, anti-inflammatory, and antibacterial characteristics, among other possible health advantages. Figure 1 shows the structure of quercetin.

Quercetin (3,3′,4′,5,7-pentahydroxyflavone) is a flavonoid and polyphenol found in high concentrations in several plants and fruits, such as capers, cranberries, figs, red onions, asparagus, radish leaves, walnuts, broccoli, and coriander. It has unique properties that enhance physical and mental performance and decrease infection risk [1]. Some naturopathic doctors use quercetin products for the treatment or management of health immunity and for inflammation. A study on such use was performed on two different groups of individuals, namely, the general population and competitive athletes. Athletes have a high risk of experiencing inflammation due to their intense exercise schedule, and this is responsible for their lower immune function as compared to the general population [2]. The name quercetin comes from “Quercetum”, which is a Latin word that means Oak Forest; it belongs to the class of flavonols, and it cannot be synthesized in the human body. It is yellow. It is not soluble in cold water, is poorly soluble in hot water, and is barely soluble in alcohol. It is one of the most abundant bioflavonoids used to treat metabolic disorders. It exerts various beneficial impacts on human health, including protection against many diseases like cardiovascular disease [3,4].

In recent decades, quercetin’s anti-inflammatory, free radical scavenging, antidiabetic, anticancer, cardiovascular, hepatoprotective, neuroprotective, antiplatelet, antibacterial, and anti-obesity properties have attracted the attention of researchers [5].

A study that aimed to treat wounds in the lower limb skin of diabetes mellitus patients using nano-hydrogel embedded with quercetin and oleic acid showed a significant reduction in wound healing time and elevation in keratinocytes, which suggests the effectiveness of this formulation in the management of wound healing [6].

Due to its poor water solubility, substantial first-pass metabolism, and consequent low bioavailability, the use of quercetin as a therapeutic molecule in clinical research is limited despite its numerous pharmacological advantages and long history of usage as a nutraceutical. To circumvent this limited bioavailability, researchers have investigated the pre-clinical and clinical use of quercetin using several drug delivery methods, such as nanoparticles (NPs), nanoemulsions, solid dispersion, micelles, and inclusion complexes [7].

In this review, we mainly focus on pharmacological action, clinical trials, patents, marketed products, and the approaches to improving the bioavailability of quercetin with the use of a nanoformulation. It is the first review of its type written about quercetin and its application regarding nanoformulations. It should be an interesting study for researchers from various backgrounds to understand quercetin pharmaceutical applications.

## 2. The Structure and Sources of Quercetin

Quercetin-type flavonols (mainly as quercetin glycosides), the most prevalent flavonoid molecules, are widespread in plants. Apples, berries, Brassica vegetables, capers, grapes, onions, shallots, tea, and tomatoes contain them, as well as many seeds, nuts, flowers, barks, and leaves. Tabulated data are shown in Table 1, showing the various sources and chemical components of quercetin. In addition, quercetin is also present in some medicinal plants, such as *Ginkgo biloba* L., *Hypericum perforatum* L., and *Sambucus canadensis* L. In red onions, larger quantities of quercetin are found in the outermost rings and in the portion of the plant that is closest to the root; this later portion has the greatest concentration [4]. The research discovered that tomatoes cultivated organically had 79% more quercetin than those grown chemically [8]. Quercetin is contained in honey derived from many plant sources. Vegetables, fruits, berries, nuts, drinks, and other products derived from plants are food-based sources of quercetin. Raw capers have the highest content, 234 mg/100 g of the edible part, while black or green tea (*Camellia sinensis* L.) has the lowest concentration of, 2 mg/100 g of the edible portion [9]. Numerous glycosidic forms of quercetin are found in plants, with quercetin 3-rutinoside, also known as quercetin-3-rhamnoglucoside or rutin, representing the most common form [10]. In onions, quercetin is associated with one or two glucose molecules (quercetin 4V-glucoside and quercetin-3,4V-glucoside). Apples contain quercetin galactosides, and berries contain quercetin arabinosides, which are examples of other dietary quercetin glycosides [11]. A diet of flavanols also has kaempferol (broccoli), myricetin (berries), and isorhamnetin (onion). Quercetin is widely distributed in the fruit, roots, and leaves of different traditional medicinal plants, including *Crataegus pinnatifida* Bunge., *Petroselinum Crispum* (Mill.) Fuss., *Nepeta cataria* L., *Polygonum orientale* L., *Mentha canadensis* L., and *G. biloba* L. Moreover, the sources of quercetin depend on the region; for example, in Japan, quercetin is mainly in onion and green tea in winter and summer, while in summer, it is available in red lettuce, green pepper, asparagus, and tomato. Whereas in Australia, quercetin is provided by black tea, green tea, coffee, apples, lettuce, green beans, grapes, tomato, lettuce, and onion [12].

### Chemistry of Quercetin and Its Derivatives

**Figure 2:**
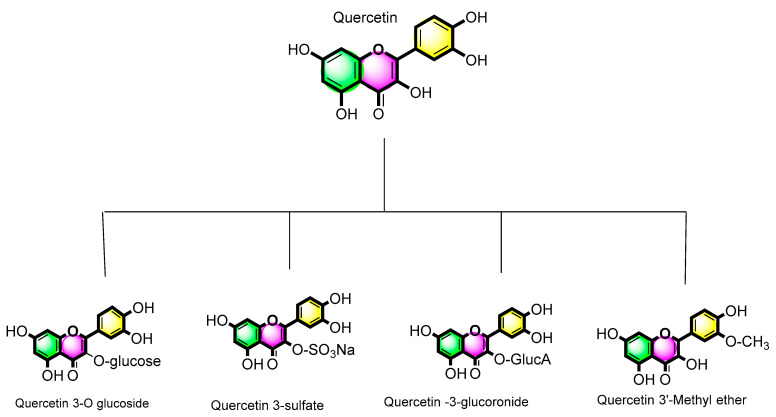
Quercetin and its derivates.

Quercetin (3,31,41,5,7-pentahydroxyflavone; Figure 2) is a naturally occurring flavonoid (flavone signifies yellow) and a flavone derivative (2-phenylchromen-4-one). Five hydroxyl groups are accountable for their biological activity and derivative diversity. Generally, flavonoids comprise two benzene rings connected by pyran or pyrone rings. The most abundant quercetin derivatives are glycosides [29,30]. O-glycosides of quercetin have O-glycosidic linkages at the C-3 carbon hydroxyl group. Additionally, an uncommon quercetin derivative form known as a C-glycoside was discovered in *Ageratina calophylla* Greene [31]. This particular C-glycoside has C-6 carbon as its glycosylation site. Another atypical derivative of quercetin, quercetin 3-O—L-fucopyranoside, was discovered in the red alga *Acanthophora spicifera* (Vahl) Børg., as well as in grapes (*V. vinifera* L.). In this form, quercetin is linked to an α-L-fucopyranosyl moiety by a glycosidic linkage at the C-3 position [32].

Strong intramolecular H-bonding is exhibited by quercetin, which helps to explain the biological multi-functionality of the compound and enables it to establish strong complexes, often with metals, which alter its bioavailability and transport [32].

Two of these H-bonds are associated with carbonyl groups, and the third one is with hydroxyl groups forming a bond between themselves. In addition to sugar, the derivative of quercetin glycosides might even comprise acyl and sulfur substituents. The hydroxyl groups of quercetin are bonded to alcohols by ether bonds in the case of ether derivatives. Despite the lipophilic nature of quercetin, the glycosylation of quercetin derivatives can boost hydrophilicity, which in turn makes it possible for the molecules to move freely throughout the plant and reach all of its tissues. Previous research has demonstrated that quercetin possesses powerful antioxidant properties, which can be linked to the presence of a hydroxyl group in the A ring and a catechol group in the B ring [33].

Because of this ability, quercetin can decrease the activities of enzymes by removing free oxygen species that are already present in the body through the transfer of hydrogen or electrons or by the chelation of metal ions. This process of scavenging reactive oxygen species helps to minimize inflammation and protects cells from the oxidative stress that can be generated by smoking or exercising for an extended period. Quercetin is also known as a lipid peroxidation inhibitor, which means that it stops the oxidation of low-density lipoproteins (LDL) and protects lipid membranes from being damaged. The α-tocopherol (primary oxidant) present in human cell membranes and LDL protects against the detrimental consequences of oxidation. Therefore, flavonoids can hinder the oxidation of lipoproteins as a result of their contribution of the hydrogen atom to the α-tocopheryl radical. In addition, it increases glutathione levels and prevents the generation of free radicals.

## 3. Pharmaceutical Applications and Activities of Quercetin

### 3.1. MOA of Quercetin in Prostate Cancer

#### 3.1.1. Inhibition of Proliferation

The processes of mitosis and cell proliferation play a significant role in the growth of tumors. As a consequence of this, arresting the cell cycle and inhibiting cell growth are two effective treatments for cancer. PC-3 human prostate cancer cells were treated with quercetin at various concentrations (50–200 μM) for 24 and 48 h, and it was observed that cell viability decreased over time. In addition, quercetin therapy increased the G2/M phase population in PC-3 and LNCaP cells and the S phase population in PC-3 cells in a dose-dependent manner [34]. It was attributed to the establishment of G0/G1 and sub-G1 cell cycle arrest, which was triggered by the downregulation of cyclin D and E, CDK2, cdc25c, and the overexpression of p21, p53, p18, and p27. PPC1 prostate cancer cells were arrested in the cell cycle, and their proliferation was slowed by a high dose of quercetin [35].

**Figure 3:**
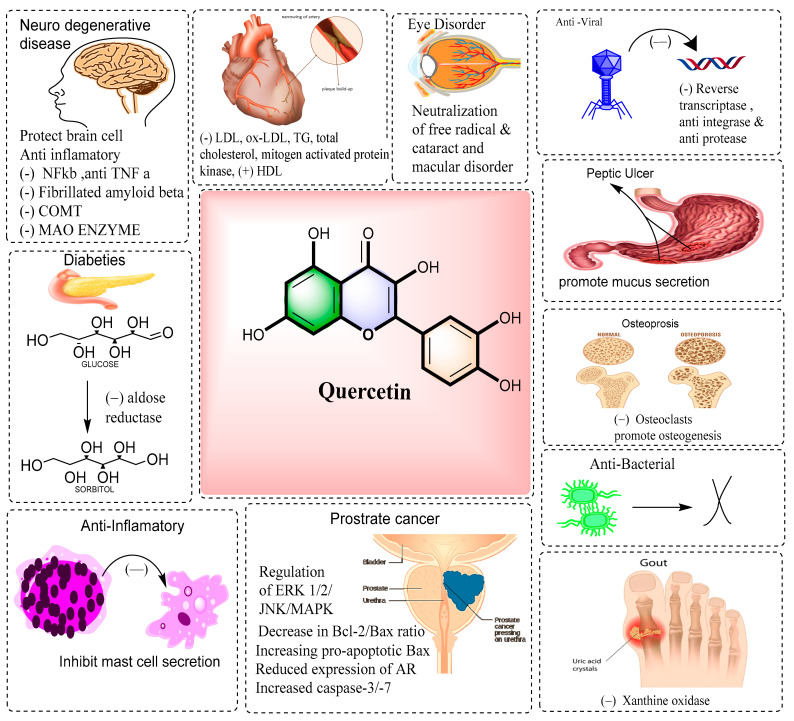
A schematic diagram represents the pharmacological efficacy of quercetin.

The endoplasmic reticulum (ER)-mediated and non-ER processes, as well as the cell cycle inhibition produced by cyclin D1 and E downregulation, were responsible for quercetin’s ability to reduce proliferation in PC-3 cells in a dose-dependent manner at an amount that was not cytotoxic [36]. Figure 3 shows a schematic diagram representing the pharmacological efficacy of quercetin. A pro-drug of quercetin (3’(N-carboxymethyl) carbomyl-3,4’, 5,7-tetrahydroxyflavone), QC12, has been used in six cancer patients. They were administered 400 mg of QC12, which is equivalent to 298 mg of quercetin; they received QC12 orally on day 1 and intravenously on day 14. In oral administration, QC12 and quercetin were not detected in the plasma, while following IV infusions. OC12 was detected in the plasma, and quercetin was detected after IV administration of QC12. The estimated bioavailability of quercetin was 20–25% released from QC12 [37].

#### 3.1.2. Induction of Apoptosis

Maintaining cellular homeostasis requires a process called apoptosis, or programmed cell death. It was categorized as a death receptor-mediated extrinsic pathway and a mitochondrial-mediated intrinsic pathway. Both of these activate the well-known executor caspase-3, which ultimately leads to the death of the cell. As insufficient apoptosis is a major contributor to carcinogenesis, numerous therapeutic medicines stimulate apoptosis. Researchers examined quercetin’s effect on PC-3 and LNCaP cells. They discovered that it induced apoptosis by increasing pro-apoptotic Bax and decreasing the anti-apoptotic Bcl-2 protein, causing a significant decrease in the Bcl-2/Bax ratio [38].

Quercetin’s effects on PC-3 cells included an increase in pro-apoptotic Bax and a decrease in anti-apoptotic Bcl-2, as well as an upsurge in ER stress-associated proteins, including GRP78, ATF-4, and IRE-1. Apoptosis was induced as a result of the mitochondrial route as well as ER stress when the caspase cascade was directly activated. Quercetin enhances extrinsic apoptosis caused by the tumor necrosis factor-related apoptosis-inducing ligand (TRAIL) in DU-145 cells through DR5 upregulation or surviving downregulation via the ERK-driven deacetylation of histone H-3 in PC-3 and DU-145 cells or the dephosphorylation of AKT in LNCaP and DU-145 cells [39].

#### 3.1.3. Inhibition of the Androgen Receptor (AR)

Androgen Receptor (AR) inhibition is a nuclear receptor that belongs to the family of ligand-responsive transcription that governs the physiological activities of androgen and is almost always expressed in prostate cancer. After treating lymph node cells (LNCaP) with quercetin for a certain period, we observed a dose-dependent decrease in AR protein. In addition, the regulating tumor indicators PSA and hK2 were suppressed, and the mRNA expression of regulated genes like PSA, NKX3.1, and ornithine decarboxylase (ODC) was reduced. These results show that quercetin has the potential to act as a chemotherapeutic agent for prostate cancer since it not only inhibits AR expression but also affects AR function [40].

**Figure 4:**
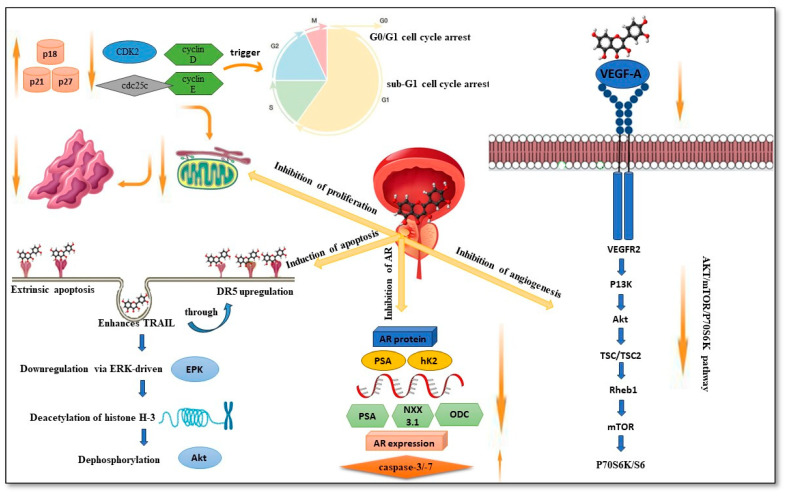
A schematic illustration of the Mechanism of action of quercetin in prostate cancer.

The treatment with quercetin decreased the expression of AR, which subsequently led to a rise in caspase-3/-7, which ultimately caused LNCaP cells to undergo apoptosis and inhibited proliferation. Additionally, it inhibited the activity of the AR protein by interacting either directly or indirectly with c-Jun or Sp1, which either formed a protein complex with AR or interacted directly with AR [41]. In addition to inhibiting DNA synthesis and modulating the AR signaling pathway, quercetin has additional anti-prostate cancer effects via AR. The general findings included lower AR expression and function, as well as the suppression of prostate cancer development [42]. Figure 4 and Table 2 show the mechanism of action of quercetin in prostate cancer.

#### 3.1.4. Inhibition of Angiogenesis

Angiogenesis is the production of new blood vessels from the existing vascular system and is controlled by two angiogenic factors: vascular endothelial growth factor (VEGF) and hypoxia-inducible factor (HIF). Tumors can produce neovascularization, and there are many blood vessels in tumor tissues. The growth assures that tumors receive sufficient oxygen and nutrients for growth and advancement. Angiogenesis is inhibited by anticancer agents that target angiogenic factors. A rhesus choroid-retina endothelial cell line (RF/6A) was treated with varied concentrations of quercetin (0–100 M), and its proliferation, migration, and tube formation were markedly decreased after 24, 48, and 72 h of incubation. These experimental findings establish that quercetin decreased in vitro angiogenesis. When PC-3 cells were treated with quercetin, VEGF production and cell survival were significantly reduced in a dose-dependent manner; this was caused by a deleterious effect on the AKT/mTOR/P70S6K pathway. Quercetin decreases angiogenesis in LNCaP cells by lowering HIF-1α accumulation and VEGF release [43].

### 3.2. Patents Related to Therapeutic Uses of Quercetin

Natural products attract the attention of researchers for their therapeutic potential with lower adverse effects compared to synthetic drugs; based on the WHO data, approximately three-quarters of the world’s population uses herbal medicine for different diseases due to their active compounds, which are attributed to their efficacy in healthcare, and many of these active ingredients are isolated and formulated in different forms such as capsules, tablets, ointment, gels, and other forms of novel drug delivery; for this reason, patent filing has increased over the past few years. Quercetin is one of the plant polyphenolic flavonols from the flavonoid group. It is extracted from many vegetables and fruits, mainly onions, broccoli, berries, grapes, cherries, and citrus fruits. Quercetin is patented alone or combined with other natural compounds for their therapeutic effects as anti-inflammatory, antiviral, antioxidant, anticancer, and cardiovascular disorders [44].

A number of patents (Table 3) have recorded quercetin derivatives as being a promising candidate in a wide range of therapeutic applications such as antioxidant, anti-inflammatory, anticoagulant, and antiaging [45]. There was a patent of quercetin recorded based on novel quercetin derivatives as an anticancer agent, suggested to be used in the preparation of drugs to minimize the risk of developing cancer; quercetin derivatives with a structure of hydrogen, benzyl, or substituted benzyl showed cytotoxic effects against cancer cell lines including ovary, lung, and prostate cancers [46]. Moreover, a quercetin derivative was synthesized from sinapinic acid, and quercetin and metal salt showed anticancer potential against human promyelocytic leukemia HL-60 cells and human myeloid leukemia cells [47]. A reaction of p-coumaric acid and quercetin synthesized another compound. The product showed anticancer activity against human tongue squamous cell carcinoma SCC-4 cells [48,49].

The 7-*O*-geranyl quercetin was suggested for its growth inhibitory effect against MCF-7 cells [50]. Another structure of quercetin derivatives that was prepared from *p*-tosyl and a methyl group exhibited high inhibitory properties against NRK-49F proliferation [51]. A quercetin derivative was synthesized using ferulic acid and quercetin as an anticancer agent, which showed a cytotoxic effect on HL-60 cells [52]. A new derivative of ferulic acid and quercetin revealed a growth inhibitory effect on HL-60 cells [53].

Some patents also showed the antioxidant effects of quercetin; a patent of 18 *α*-glycyrrhetinic, hederagenin, and quercetin showed an antiaging effect on the skin, reducing the intracellular oxidative load and stimulation of the proteasome and cell whitening [54]. Another invention method was designed to enhance kidney function by consuming a composition of quercetin, vitamin B3, and folic and vitamin C; this combination can be administered as a beverage, jelly, paste, tablet, or capsule [55]. Another invention uses sinapinic acid, quercetin dehydrate, and a metal salt. The study evaluated the anticancer effect of this combination against human monocytic leukemia cells (THP-1) and HL-60 cells [56].

A patent synthesized a quercetin derivative containing benzylpiperazine; this composition showed a protective effect against gastric mucosal lesions due to its anti-inflammatory and antibacterial activity [57,58]. A patent was reported for treating thrombotic disorders using quercetin along with folic acid, vitamin C, and vitamin B3 [59].

**Table 3:** Patents related to therapeutic uses of quercetin.

S. No.	Patent Publication Number	Title of the Patent	Activity and Application	Reference
1.	US20040161247A1	Therapeutic agent for osteoporosis comprising an active ingredient of quercetin derivatives	The present invention relates to a therapeutic agent for osteoporosis, which comprises an active ingredient of quercetin derivatives.	[60]
2.	US8440704B2	Quercetin-containing compositions	This invention relates to a composition containing quercetin, vitamin B3, and vitamin C.	[61]
3.	JP3896577B2	Quercetin glycoside composition and preparation method	Preparation of compounds containing saccharide radicals produced by the action of a beta-amylase, e.g., maltose.	[62]
4.	AU2012340840B2	Method for treating hepatitis C virus infection using quercetin-containing compositions	Heterocyclic compounds having nitrogen as a ring hetero atom, e.g., guanethidine.	[63]
5.	WO2009045735A2	Method for stabilizing quercetin	This invention relates to a method for stabilizing quercetin by placing it in a solution containing vitamin B3 and vitamin C and assessing the stability of the quercetin in the mixture.	[64]
6.	CA2735826C	Reducing cholesterol levels with combined use of quercetin and statin	The use of a first composition containing quercetin, vitamin C, and vitamin B3, and a second composition containing a statin and a pharmaceutically acceptable carrier, for reducing the plasma cholesterol level in a subject is described.	[65]
7.	CN103145669A	Process of clean production of quercetin	Process of clean production of quercetin.	[66]
8.	US6562866B1	Method for preventing or treating elevated blood lipid level-related diseases by administering rutin and quercetin	Method for treating or preventing an elevated blood lipid level-related disease in a mammal, which comprises administering thereto an effective amount of rutin, quercetin, or a mixture thereof.	[67]
9.	US5445842A	Quercetin-containing coloring	A quercetin-containing colorant that has, as an effective component, a quercetin that is included by cyclodextrin.	[68]
10.	CN101111244B	Composition for promoting production of hyaluronic acid containing kaempferol and quercetin	Disclosed is a composition for promoting the production of hyaluronic acid containing at least one of kaempferol and quercetin.	[69]
11.	CN104817603A	Method for preparing quercetin-3-O-beta-D-glucuronide from lotus seedpod	The invention relates to a method for preparing quercetin-3-O-beta-D-glucuronide from lotus seedpod.	[70]
12.	US7049301B2	Quercetin derivatives and their medical usages	This invention relates to quercetin derivative, its preparation, and a pharmaceutical combination, as well as their medical uses for the prevention or treatment of diseases.	[71]
13.	US9289444B2	Composition for promoting hematogenesis containing quercetin 3-O-β-(2″-galloyl)-rhamnopyranoside as active ingredient	This invention relates to a composition for promoting hematopoiesis and for treating, preventing, or alleviating cytopenia or bone marrow failure comprising quercetin 3-O-β-(2″-galloyl)-rhamnopyranoside (QGR) as active ingredient.	[72]
14.	CN101955514A	Method for synthesizing agarose gel hydrogen bond adsorbing chromatography medium by using quercetin as genin	The invention relates to a method for synthesizing a hydrogen bond adsorbing medium by taking agarose gel as a matrix and taking quercetin as genin.	[73]
15.	TWI618573B	A quercetin-type surfactant, its preparation method and application.	The invention prepares a natural quercetin-type surfactant and uses the quercetin and the diol compound to synthesize a quercetin derivative under an acidic catalyst.	[74]
16.	US20080031940A1	Quercetin-containing composition, methods of making, and methods of using	The composition is a sustained release composition in tablet or capsule form suitable for oral administration to a human. Methods of making and using the composition are provided.	[75]
17.	RU2745123C1	Bioactive composition based on a cross-linked hyaluronic acid salt containing resveratrol and a method of its preparation a method for its preparation	Bioactive composition based on a cross-linked hyaluronic acid salt containing quercetin and a method for its preparation.	[76]
18.	CN106822085B	Application of oncolytic adenovirus expressing TRAIL and quercetin in inhibition of liver cancer cell proliferation	Application of oncolytic adenovirus expressing TRAIL and quercetin in inhibition of liver cancer cell proliferation.	[77]

### 3.3. Clinical Trials of the Therapeutic Effects of Quercetin

#### 3.3.1. Cardiovascular Disease (CVD) Risk

A double-blind, randomized clinical trial was carried out for 10 weeks on 72 women. The subjects were given 500 mg of quercetin daily. Quercetin significantly decreased tumor necrosis factor-α (TNF-α), interleukin-6 (IL-6), high-density lipoprotein cholesterol (HDL-C), and systolic blood pressure (SBP), while low-density lipoprotein cholesterol (LDL-C), total cholesterol, and triglycerides (TG) were not significantly reduced. The results showed that quercetin supplementation did not affect cardiovascular risk factors [78]. A detailed review of quercetin and it application for cardiovascular diseases was recently conducted showed a beneficial effect on glycemic and lipid parameters [79]. In another study, healthy candidates who had 4.0–7.2 mmol/L cholesterol levels were administered 1 g quercetin daily for 28 days. There was no alteration in the cardiovascular risk factors, including HDL, LDL, and triglyceride levels. Also, no modification was noticed in the thrombogenic risk parameters, including blood pressure and platelet thromboxane B2 production [80].

A meta-analysis and systemic review of randomized controlled trials (RCTs) was used to evaluate the efficacy of quercetin in inflammatory markers and lipid profiles with patients with metabolic syndrome and related disorders; 16 RCTs were employed in the meta-analysis. The pooled results found that quercetin supplements significantly decreased total cholesterol, LDL, and C-reactive protein. In contrast, there was no significant difference found in TG, HDL, interleukin 6 (IL-6), and tumor necrosis factor-alpha (TNF-α) levels [81]. Additionally, a double-blinded, placebo-controlled cross-over trial was assigned with ninety-three overweight subjects with metabolic syndrome to examine the effect of quercetin consumption on lipid metabolism, blood pressure, inflammation, body consumption, and oxidative stress markers. The participants received 150 mg of quercetin for 6 weeks. It was found that quercetin reduced SBP by 2.6 mmHg, and it also decreased HDL; in contrast, there was no change noticed in TAG, total cholesterol, and the LDL:HDL and TAG:HDL ratios. Quercetin reduced atherogenic oxidized LDL (ox-LDL) levels, but no alteration was determined in C-reactive protein and TNF-α compared with the placebo group. There were no adverse effects on the kidney and liver functions, which provides evidence that quercetin has a beneficial role as a preventive measure against CVD [82].

A randomized, double-blinded, placebo-controlled parallel design was assigned randomly into two groups: placebo group (*n* = 43) and 100 mg quercetin (n = 49) for 10 weeks. It revealed that quercetin decreased total cholesterol, LDL, systolic, diastolic blood pressure, and glucose concentrations significantly. Furthermore, it increases HDL. In contrast, no effects of quercetin were found for the inflammatory markers, sVCAM-1 and IL-6 [83]. Furthermore, a double-blind cross-over study with 49 healthy candidates consumed 150 mg placebo or quercetin for 8 weeks, intermitted by a washout phase for 3 weeks. Quercetin reduced postprandial triacylglycerol, postprandial SBP, and waist circumference, and it increased HDL and TNFα [84]. In addition, a double-blind, randomized study was designed to study the effect of quercetin on blood lipid values among healthy persons with dyslipidemia. After two months of the treatment, a decrease in triglycerides, cholesterol (from 6.21 mmol/L to 5.09 mmol/L), and LDL (from 3.98 mmol/L to 2.91 mmol/L) and an increase in HDL (from 0.89 mmol/L to 1.29 mmol/L) were demonstrated. These findings agree that quercetin is a promising candidate for lowering blood lipids [85].

In a study conducted with 48 hyper-cholesterolemic and hypertensive subjects with statin intolerance, the participants received 10 mg of quercetin combined with ezetimibe daily for 3 months. The findings showed a reduction in TC and LDL levels while there were no changes were observed in TG and HDL [86]. Data from 18 RCTs, looking at the response in blood lipids of subjects who were given supplementation with flavonols, particularly quercetin, revealed a notable decrease in the TC, LDL, and TG; additionally, a significant elevation was observed in HDL, whereas fasting plasma glucose and BP were significantly reduced [87]. A randomized double-blind placebo-controlled parallel-group study was performed using 70 healthy subjects who consumed quercetin-rich onion for 12 weeks; a reduction in LDL levels in the candidates whose LDL was higher than normal was observed [88]. Egret and group conducted, a double-blind placebo-controlled cross-over study for evaluating the effect of 3.6 g/d alpha-linolenic acid (ALA) plus 190 mg/d quercetin for 8 weeks for 67 subjects. They reported that with ALA with or without quercetin in healthy non-obese adults resulted in significant improvements in fasting serum LDL-C, non–HDL-C, and Apolipoprotein B100 [89].

#### 3.3.2. Anti-Inflammatory

A combination of 1000 mg of quercetin and 100 mg of Dasatinib was administered orally for patients with diabetic kidney disease; the results showed a reduction in adipose tissue senescent cell burden within 11 days, which led to minimizing the release of dysfunction and inflammation factors. It also reduced senescence-associated secretory phenotype (SASP), which accumulates at etiological sites in chronic diseases, including MMPs-9, MMPs-12, IL-1α, and IL-6 [90]. In another study with eighty-four patients who were divided into two groups for 12 weeks, 42 subjects were given 500 mg/day quercetin tablet, and 42 subjects were given 500 mg/day starch placebo. Quercetin reduced iron, ferritin, transferrin saturation, and high sensitivity C-reactive protein while it increased transferrin, which makes it a potent agent in improving the iron status in thalassemia. It was also found that quercetin had no significant effect on tumor necrosis factor-α (TNF-α) and total iron binding capacity, so it can be concluded that quercetin is indistinctive on inflammation [91]. In addition, a meta-analysis study was conducted to evaluate the anti-inflammatory potential of quercetin; it revealed a significant effect on the C-reactive protein (CRP), specifically if it is taken at doses above 500 mg/day in patients with o3 mg/L CRP [92]. Furthermore, in a randomized, double-blind, placebo-controlled clinical trial in 50 women with rheumatoid arthritis (RA), they were assigned to a placebo group and a quercetin (500 mg/day) group for 8 weeks. Quercetin was found to reduce morning pain, after-activity pain, and early morning stiffness (EMS); high-sensitivity tumor necrosis factor-α (hs-TNF α) was also significantly reduced, while there were no significant differences in erythrocyte sedimentation rate (ESR), swollen (SSC), and tender joint counts (SJC) [93].

#### 3.3.3. Pulmonary Diseases and COVID-19

Supplementation of quercetin was given to chronic obstructive pulmonary disease (COPD) patients, it was given for one week in a dose escalation manner of 500, 1000, or 2000 mg/day. The results showed that quercetin administration was safe up to 2000 mg/day as evaluated by blood profile and lung function questionnaire [94]. Moreover, a randomized, double-blinded, placebo-controlled trial measured the effect of (500 and 1000 mg/day) quercetin compared to placebo in 1002 patients with upper respiratory tract infection (URTI) rates for 12 weeks. It was shown that there was no significant effect of quercetin on URTI rates as compared to the placebo [95]. Moreover, an open-label, randomized controlled clinical trial was conducted to evaluate the effect of curcumin and quercetin with daily doses of 168 mg and 260 mg, respectively; the treatment was given to 25 patients in early to mild-stage COVID-19 at home twice a day. The finding exhibited a speedy resolution in the treated patients [96].

Similarly, a study examined the efficacy of quercetin in 60 severe cases of hospitalized COVID-19 patients for 7 days with 1000 mg of quercetin along with antiviral drugs. The study showed the effectiveness of quercetin in reducing LDH, q-CRP, and ALP levels, in addition to increasing the respiratory rate and hemoglobin level [97]. In addition, an open-label randomized controlled clinical trial was conducted to assess the influence of 500 mg of quercetin for two weeks in 50 outpatients with early to moderate stages of COVID-19. In the first week, patients in the quercetin group showed a speedy recovery from COVID-19; 34 patients tested negative in the treated group, while 12 patients tested negative in the control group; the patients who received quercetin showed a significant reduction in lactate dehydrogenase (LDH) level [98].

A randomized, open-label, and controlled clinical trial was recruited to 42 COVID-19 outpatients. Twenty-one patients were subjected to standard-of-care treatment, and twenty-one patients were subjected to add-on quercetin supplementation. After 1 week of treatment, 16 patients of the quercetin group tested negative, and 12 patients showed a diminishment in all their symptoms; in the standard of care group, 2 patients tested negative, and 4 patients showed improvement in their symptoms. After 2 weeks, the remaining 5 patients in the add-on quercetin therapy group tested negative, while the standard-of-care group recorded 17 tested negatives out of 19 remaining patients; in the third week, 1 tested negative and 1 was still positive; and by day 20, the last patient tested negative. In terms of blood parameters, the add-on treatment with quercetin decreased D-dimer, LDH, CRP, and ferritin levels. It can be concluded that add-on quercetin supplementation can reduce the severity and speed the recovery of COVID-19 patients [99].

#### 3.3.4. Polycystic Ovary Syndrome (PCOS)

A randomized clinical trial was examined with 84 women having PCOS; the subjects were assigned to two groups; the treated group was administered 1 g of quercetin daily for 12 weeks while the control group received a placebo. It was shown that quercetin supplements increased the expression of the *ADIPOR1* and *ADIPOR2* transcript; it also improved the AMPK level by 12.3% when compared to the control group [100]. Another study aimed to detect the effect of quercetin on adiponectin-mediated insulin sensitivity in Polycystic ovary syndrome (PCOS) patients. For this purpose, eighty-four women with PCOS were enrolled into two groups; the treatment group received daily 1 g quercetin in the form of two 500 mg capsules for 12 weeks, whereas the control group took a placebo. Quercetin slightly elevated the adiponectin level by 5.56% as compared to the placebo; it also increased high-molecular-weight (HMW) adiponectin by 3.9% when compared to the placebo, while it reduced the level of luteinizing hormone (LH) and testosterone. It was also recorded that quercetin significantly lowered Homeostatic Model Assessment for Insulin Resistance (HOMA-IR) levels by 1.84 compared to 2.21 in the placebo group. Oral quercetin was effective in enhancing adiponectin-mediated insulin resistance and improving the hormonal profile of women with PCOS [101].

A systematic review was carried out to evaluate the therapeutic activity of quercetin in women with PCOS. Out of 253 articles that were implemented for the study, there were 8 articles eligible; 3 of them are clinical trials and 5 animal studies, and the studies reported that quercetin is able to reduce LH, testosterone, and insulin resistance. Despite the fact that quercetin enhanced dyslipidemia, there was no significant difference in weight loss, suggesting that quercetin has anti-inflammatory and antioxidant features rather than weight-reducing effects. Thus, this review is evidence of quercetin’s potential to attenuate PCOS complications [102].

#### 3.3.5. Diabetes Mellitus

A randomized blinded cross-over study was carried out to determine the influence of quercetin on postprandial hyperglycemia in Type 2 Diabetes Mellitus (T2DM) patients. It was revealed that 400 mg of quercetin was able to reduce postprandial hyperglycemia in T2DM patients effectively [103]. Another clinical trial aimed to assess the antioxidant effect of 250 mg of quercetin daily; the study assigned forty-seven patients with T2DM for 8 weeks. It was proven that quercetin significantly ameliorated the TAC compared to the placebo group. A reduction in serum concentration of ox-LDL was also detected. However, there was no effect on glycemic parameters, including serum insulin, FBS, and glycosylated Hb (HbA1c) levels. Also, no alteration was determined in the lipid profile, so it can be concluded that quercetin has a beneficial effect on antioxidant status with no effect on glycemic and lipid status [104,105]. Furthermore, a meta-analysis and systematic review was performed using randomized controlled trials to investigate the effect of quercetin on glycemic control among patients with metabolic syndrome. Nine studies were employed and were eligible for the study; it was found that quercetin reduced FPG with ≥8 weeks of the treatment with a dosage of ≥500 mg/day, with a significant reduction in insulin concentrations at a dosage of ≥500 mg/day [106].

#### 3.3.6. Obesity

A randomized, double-blind, placebo-controlled study was performed on overweight subjects; the subjects were assigned to take either the placebo, which was 6 males and 30 females, or 100 mg quercetin, which was 5 males and 31 females, for 12 weeks. Quercetin significantly decreased the weight and percentage of body fat compared to the control group, while the blood glucose and leptin levels were reduced in both control and treated groups [107]. In contrast, a meta-analysis study was conducted using excellent, relevant, randomized controlled clinical trials (RCTs) with 525 participants to evaluate the effect of quercetin on weight loss, with no significant effects found on body weight, body mass index, waist circumference, or waist to hip ratio, which confirms that there is no notable effect of quercetin on weight loss [108].

#### 3.3.7. Antioxidant

Oral supplantation of 500 mg/day quercetin for 8 weeks in post-myocardial infarction patients significantly increased TAC and decreased TNF-α levels, and it improved the insecurity dimension of quality of life [109].

#### 3.3.8. Blood Pressure

A double-blind, placebo-controlled, cross-over study was conducted using 19 subjects with prehypertension, and 22 subjects with hypertension were assigned to examine the effect of 730 mg quercetin for 28 days. It was shown that blood pressure did not change in prehypertensive subjects; on the other hand, there was a notable reduction in the systolic and diastolic blood pressure in the hypertension subjects after quercetin supplantation, although no effect was detected in oxidative stress [110]. There were 17 RCTs in which 896 subjects were included in the analysis. A dose of 30–1000 mg quercetin was given daily for 2–12 weeks. SBP and DBP were significantly reduced. Another change was observed in HDL and TG when candidates were given quercetin for 8 weeks or more [111].

#### 3.3.9. Other Therapeutic Effects

A study was conducted using Bushen, Yiqi, Lixue, and Yangtai (BYLY), which is a traditional Chinese quadri-combination therapy used to treat recurrent spontaneous abortion (RSA); BYLY has 132 active components, and quercetin might be the key effective component. In this regard, four hundred and eighty participants were assigned to the clinical trial, it was shown that combined duphaston and BYLY could decrease the rate of early abortion compared with the use of duphaston or BYLY alone. It is believed that a possible mechanism is the protective effect of quercetin on trophoblasts by decreasing Drp1 expression through regulating miR-34a-5p [112]. In another randomized, double-blind, placebo-controlled trial using quercetin for two weeks, the results revealed that alanine aminotransferase (ALT), aspartate aminotransferase (AST), and gamma-glutamyl transferase (GGT) were decreased by 50.4%, 37.2%, and 89.9%, respectively. In addition, the levels of TG, TC, and TNF-α reduced by 33.3%, 16.7%, and 39.8%, respectively, suggesting the potential value of quercetin in treating non-alcoholic fatty liver disease (NAFLD). Also, ferritin was reduced, and RBC increased significantly [113]. In Table 4, a list of clinical trials of the therapeutic effects of quercetin is recorded.

Another double-blind, placebo-controlled randomized trial was conducted to determine the influence of quercetin on 20 adult patients who received high-dose chemotherapy for the treatment of blood malignancies. Patients were subjected to two groups (10 patients in the control group and 10 patients in the intervention group). Patients in the treated group received 250 mg quercetin capsules twice daily for 4 weeks. It was concluded that the incidence of oral mucositis was lower in the quercetin group (three patients in the intervention group and six patients in the control group). Still, the severity of oral mucositis was higher in the treated group compared to the control group (2.6 vs. 2, respectively), which might be due to lower oral health care in the intervention group [114].

**Table 4:** Clinical trials of the therapeutic effects of quercetin.

Therapeutic Effect	Design of the Study	Number of Subjects	Dosage of Quercetin	Duration	Results	Reference
Antioxidant	RCT	NA	500 mg/day	8 weeks	It significantly increased antioxidant capacity (TAC) and decreased TNF-α levels and it improved the insecurity dimension of quality of life.	[115]
Anti-inflammatory	RCT	100	500 mg	9 days	Quercetin supplementation may help limit the vigorous inflammatory response triggered by coronary artery bypass (CABG) and subsequent postoperative complications in patients suffering from an acute coronary syndrome.	[116]
	A meta-analysis study of RCTs	NA	500 mg/day	NA	It showed a significant effect on CRP.	[92]
	RCT	NA	>500 mg/day	8 weeks	It reduced morning pain, after-activity pain, and early morning stiffness (EMS); hs-TNFa was significantly reduced while there were no significant differences in ESR, swollen (SSC), and tender joint counts (SJC).	[93]
	RCT	42	500 mg/day	12 weeks	It reduced iron, ferritin, transferrin saturation, and high sensitivity CRP while it increased transferrin, which makes it a potent agent in improving iron status in thalassemia. It was also found that quercetin had no significant effect on TNF-α and total iron binding capacity.	[91]
Obesity	Meta-analysis study of RCTs	525 subjects	NA	NA	No significant effect on body weight, body mass index, waist circumference, and waist-to-hip ratio, which confirms that there is no noticeable effect of quercetin on weight loss.	[108]
	Randomized, double-blind, placebo-controlled study	37 female	100 mg capsule	12 weeks	Quercetin-rich onion peel extract (OPE) supplementation significantly decreased the percent of body fat mass (PBFM) and induced plasma adiponectin levels compared with baseline values in addition also reduces the percentage of, BMI, and waist circumference, which corresponds to the results of this study.	[117]
	Double-blind cross-over study	49 healthy candidates	150 mg/day	8 weeks, intermitted by washout phase for 3 weeks.	It reduced postprandial triacylglycerol, postprandial SBP, and waist circumference while increasing HDL and TNFα.	[92]
Pulmonary diseases and COVID-19	RCT	Chronic obstructive pulmonary disease patients	500, 1000, or 2000 mg/day	1 week	It showed that quercetin administration was safe up to 2000 mg/day as evaluated by blood profile and lung function questionnaire.	[94]
	Open-label, randomized controlled clinical trial	25 patients in early to mild stage of COVID-19	168 mg and 260 mg	NA	It exhibited a speed resolve in the treated patients.	[96]
	RCT	60 severe cases hospitalized COVID-19	1000 mg/d	1 week	It showed the effectiveness of quercetin in reducing LDH, q-CRP, and ALP levels, in addition to an increase in the respiratory rate and hemoglobin level.	[97]
	Open-label randomized controlled clinical trial	50 outpatients with early to moderate stage of COVID-19	500 mg/d	2 weeks	It showed a speedy recovery from COVID-19 as 34 patients tested negative in the treated group while 12 patients tested negative in the control group; the patients who received quercetin showed a significant reduction in lactate dehydrogenase (LDH) level.	[98]
	Randomized, open-label, and controlled clinical trial	21 outpatients of COVID-19	NA	2 weeks	The add-on treatment with quercetin, decreased D-dimer, LDH, CRP, and ferritin levels. It can be concluded that add-on quercetin supplantation can reduce the severity and speed the recovery in COVID-19 patients.	[99]
	Randomized, double-blinded, placebo-controlled trial	1002 patients with upper respiratory tract infection URTI	500 and 1000 mg/day	12 weeks	It was shown that there is no significant effect of quercetin on URTI rates as compared to placebo.	[95]
Polycystic Ovary Syndrome (PCOS)	RCT	84 women with PCOS	1 g of quercetin daily for	12 weeks	It was shown that quercetin supplements increased the expression of *ADIPOR1* and *ADIPOR2* transcript, and it also improved AMPK level by 12.3% when compared to the control group.	[100]
	RCT	84 women with PCOS	1 g	12 weeks	It slightly elevated adiponectin level by 5.56% as compared to placebo, and it also increased HMW adiponectin by 3.9% when compared to the placebo, while it reduced the level of LH and testosterone. It was also recorded that quercetin significantly lowered HOMA-IR levels by 1.84 compared to 2.21 in the placebo group.	[101]
	A systematic review of 3 RCTs	NA	NA	NA	It reported that quercetin was able to reduce LH, testosterone, and insulin resistance. Despite the fact that quercetin enhanced dyslipidemia, there was no significant difference in weight loss, suggesting that quercetin has anti-inflammatory and antioxidant features rather than weight-reducing effects.	[102]
Diabetes Mellitus	Randomized blinded cross-over study	NA	400 mg	NA	It was revealed that 400 mg of quercetin was able to reduce postprandial hyperglycemia in T2DM patients effectively.	[118]
	RCT	47 patients with T2DM	250 mg/day	8 weeks	It enhanced TAC significantly compared to cellulose placebo group, and it also showed a notable reduction in ox-LDL.	[105]
	A meta-analysis and systematic review of 9 RCTs	NA	≥500 mg/day	≥8 weeks	it was found that quercetin reduced FPG with ≥8 weeks of the treatment with a dosage of ≥500 mg/day, besides a significant reduction in insulin concentrations in a dosage of ≥500 mg/day.	[106]
Cardiovascular risk	Double-blind randomized clinical trial	72 women	500 mg of quercetin daily	10 weeks	It significantly decreased TNF-α, IL-6, HDL, and systolic blood pressure while LDL, total cholesterol, and TG were not significantly reduced.	[79]
	RCT	24 subjects	100 mL of quercetin-rich onion juice daily	8 weeks	It was proved that onion juice effectively reduced LDL-c and total cholesterol and increased TAC and also increased the lagtime of LDL oxidation. Suggesting that quercetin-rich onion juice might provide a markable effect on cardiovascular diseases.	[119]
	A meta-analysis and systemic review of 16 RCTs	NA	NA	NA	The pooled results found that quercetin supplements significantly decreased total cholesterol, LDL, and CRP. While there is no significant difference found in TG, HDL, IL-6, and TNF-α levels.	[82]
	RCT	Healthy candidates with 4.0–7.2 mmol/L cholesterol level	1 g	28 days	There was no alteration in the cardiovascular risk factors including HDL, LDL, and triglyceride levels. Also, no modification was noticed in the thrombogenic risk parameters including blood pressure and platelet thromboxane B2 production.	[81]
	Double-blind, randomized study was	Healthy persons with dyslipidemia	NA	2 months	It decreased in TG, cholesterol (from 6.21 mmol/L to 5.09 mmol/L), and LDL (from 3.98 mmol/L to 2.91 mmol/L) and increased in HDL (from 0.89 mmol/L to 1.29 mmol/L). These findings agree that quercetin is a promising candidate for lowering blood lipids.	[85]
	Double-blinded, placebo-controlled cross-over trial	33 overweight subjects with metabolic syndrome	150 mg/day	6 weeks	It was found that quercetin reduced SBP by 2.6 mmHg. It also reduced atherogenic oxidized LDL.	[82]
	Randomized, double-blinded, placebo-controlled study	49 subjects	100 mg/day	10 weeks	It revealed that quercetin decreased total cholesterol, LDL, systolic, diastolic blood pressure, and Glucose concentrations significantly. Furthermore, it increased HDL.	[83]
	RCT	530 healthy individuals	16–1200 mg	2–12 weeks	It showed a notable decrease in TC, LDL, and TG; additionally, a significant elevation was observed in HDL.	[87]
	Randomized double-blind placebo-controlled parallel-group	70 healthy subjects	3.12–3000 mg	12 weeks	A reduction in LDL levels in the candidates whose LDL was higher than normal was observed.	[88]
	Double-blind cross-over	49 healthy males	150 mg	8 weeks	The study revealed an increase in HDL, postprandial triacylglycerol, and TNFα levels.	[89]
Blood pressure	Double-blind, placebo-controlled, cross-over study	19 subjects with prehypertension, and 22 subjects with hypertension	730 mg/d	4 weeks	It was shown that blood pressure did not change in prehypertensive subjects. On the other hand, there was a notable reduction in the systolic and diastolic blood pressure in the hypertension subjects after quercetin supplantation, although no effect was detected in oxidative stress.	[110]
	RCT	896 subjects	30–1000 mg	2–12 weeks	SBP and DBP were significantly reduced. Another change was observed in HDL and TG in which candidates were given quercetin for 8 weeks or more.	[111]
	Randomized, double-blind, placebo-controlled cross-over trial	37 nonsmoking healthy adults with SBP between 125 and 160 mm Hg	160 mg/day	4 weeks	Quercetin might play a key role in the treatment of diseases in which Methylglyoxal (MGO) plays a pivotal role.	[120]
Other therapeutic effects	Randomized, double-blind, placebo-controlled trial	NA	NA	2 weeks	It was revealed that ALT, AST, and GGT were decreased by 50.4%, 37.2%, and 89.9%, respectively. The levels of TG, TC, and TNF-α reduced by 33.3%, 16.7%, and 39.8%, respectively, suggesting the potential value of quercetin in treating Non-alcoholic fatty liver disease (NAFLD).	[112]
	RCT	480 participants	NA	NA	BYLY has 132 active components, and quercetin might be the key effective component that could decrease the rate of early abortion. It is believed that a possible mechanism is the protective effect of quercetin on trophoblasts by decreasing Drp1 expression through regulating miR-34a-5p.	[111]
	Double-blind, placebo-controlled randomized trial	20 adult patients who received high-dose chemotherapy for the treatment of blood malignancies	250 mg capsules twice daily	4 weeks	It was concluded that the incidence of oral mucositis was lower in the quercetin group (3 patients in the intervention group and 6 patients in the control group), but the severity of oral mucositis was higher in the treated group compared to the control group (2.6 vs. 2. respectively), which might be due to lower oral health care in the intervention group.	[113]

### 3.4. Marketed Products

Quercetin has been widely used in dietary supplement and pharmaceutical applications, as given in Table S1 (as Supporting Information). It was recorded that the average consumption of quercetin from food is 16–25 mg/day. It is available as a nutritional supplement in tablet and powder forms with daily dosages of 500 to 1000 mg [121]. Quercetin has a tolerability and safety profile in humans. It received the status of Generally Recognized as Safe (GRAS) from the United States Food and Drug Administration (USFDA) for its use as a dietary supplement (US Food and Drug Administration Letter, 2010). Table S2 (as Supporting Information) depicts commercial products of quercetin available in the market as OTC and prescribed medicine.

## 4. Approaches for Improving Quercetin Pharmacokinetics Quercetin-Based Nanoformulation

### 4.1. Solid Lipid Nanoparticles

SLNs are first-generation lipid nanocarriers. They are colloidal carriers containing biocompatible and biodegradable lipids with a high melting point such as a solid particle that is stabilized by surfactants. They are used to formulate drugs in solid lipids by a cold or hot homogenization technique, based on the thermal stability of the drug. SLNs are usually prepared by O/W nanoemulsion at a temperature higher than the melting point of the lipid phase and then reducing the temperature to induce lipid crystallization [122,123].

The World Health Organization and USFDA permit edible solid lipids that are used as carriers, and the drug molecules are encapsulated to obtain the solid lipid nanoparticles. As a type of submicron particulate drug delivery system, SLNs possess high bioavailability, high biocompatibility, and controlled release, with various routes of administration, including intravenous, oral, transdermal, and pulmonary. SLNs can also overcome the challenges associated with oral delivery of bioactive substances that have low solubility, poor permeability, and P-glycoprotein-mediated efflux issues. SLNs were used to improve the gastrointestinal absorption of quercetin, and it was recorded that the bioavailability of quercetin-loaded SLNs was 5.71-fold greater than that of the quercetin-loaded suspension in 4% CMC-Na (sodium carboxy methyl cellulose) in rats [124]. Bose and his group developed a solvent-free solid-lipid-based nanosystem, which was identified for topical delivery of quercetin, and in vitro release research has revealed the biphasic release of quercetin from an SLN formulation, with an initial burst release followed by a prolonged release for up to 24 h [125].

Quercetin is a highly lipophilic molecule that can be conveniently combined with an SLN structure, and it was recorded that the entrapment efficiency was 100% for all formulations. A study reported that the IC50 of quercetin in cholesterol SLNs was approximately six times less than free quercetin. Phytosterol-loaded SLN was found to be cell toxic. Hence, it was documented that cellular penetration of quercetin was improved by sterol-containing solid lipid nanoparticles for targeting hepatocellular carcinoma cells [126].

### 4.2. Nanostructured Lipid Carriers

**Figure 5:**
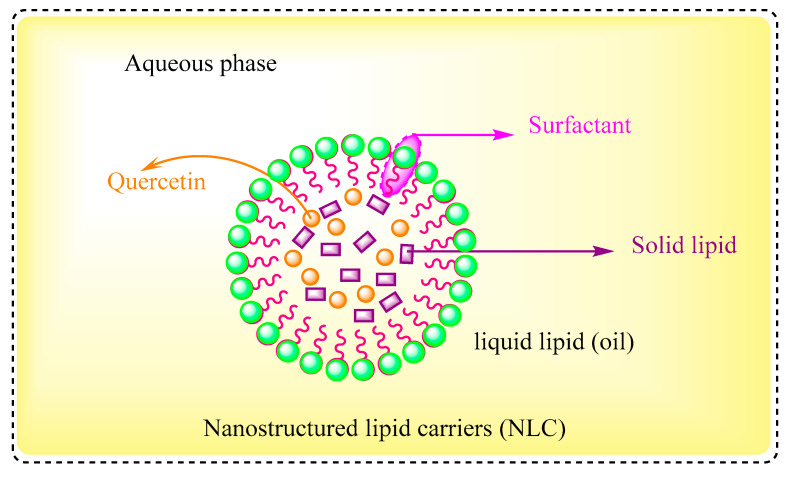
Nanostructured lipid carriers (NLCs): a mixture of oil and solid dispersed in aqueous sol containing surfactant.

Nanostructured lipid carriers (NLCs) (Figure 5) are combined from a mixture of dispersed solids and liquid lipids (oils) in an aqueous solution containing a surfactant [127]. Second-generation lipid carriers, among them NLCs, were developed in order to address the drawbacks of first-generation lipid nanocarriers, such as SLN, which included minimized drug loading efficiency and drug escape through the matrix while storage. Currently, NLCs are considered potent drug carriers and have superior formulation properties over SLNs due to their biocompatibility. NLCs demonstrated an encapsulation efficiency of >90% [128]. A study showed the retention of quercetin in the dermis and epidermis; its penetration, anti-inflammatory, and antioxidant properties were improved by loading it into NLCs, which shows that NLCs are efficient carriers for the topical delivery of quercetin [129].

Quercetin application in cancer is limited due to its poor water solubility, poor cellular bioavailability, and high instability, and to overcome this issue, researchers have used a strategy to encapsulate quercetin into biodegradable and biocompatible nanoparticles. Wang and co-authors synthesized biocompatible and biodegradable quercetin- nanostructured lipid carriers (quercetin-NLCs) by using the phase inversion-based process method. They found that quercetin-NLCs possessed good thermal stability and a sustained release pattern. The study also indicated that the quercetin-NLCs enhanced the solubility of quercetin in water by at least 1000-fold, and the activity to inhibit breast cancer was significantly improved [130].

Quercetin-NLCs significantly increased cytotoxicity in a dose-dependent manner and induced apoptosis in MCF-7 and MDA-MB-231 breast cancer cells [131]. This novel formulation of quercetin with nanostructured lipid carriers has over three times the dose benefit of indigenous quercetin to decrease the viability of breast cancer cells. It was concluded that the quercetin-NLC formulation can be a potential breakthrough for the treatment of breast cancer with minimal side effects [132].

### 4.3. Liposomes

**Figure 6:**
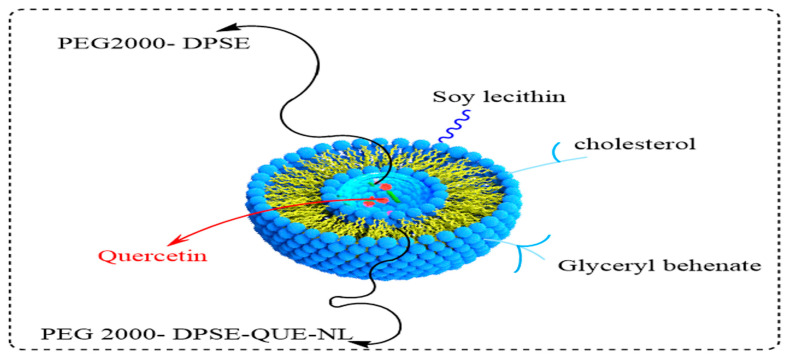
Liposomes: biocompatible spherical vehicle with a main structure consisting of natural phospholipid (soybean phosphatidylcholine/egg/dipalmitoyl phosphatidyl chlorine (DPPC)).

Liposomes are microscopic vesicles consisting of an outer layer made up of phospholipids in a bilayer, wrapping an aqueous core. Natural phospholipids or lipids, such as soybean or egg phosphatidylcholine dipalmitoylphosphatidylcholine (DPPC), are similar to those found in biological membranes. They are biocompatible spherical vehicles with a size of 80–300 nm, can entrap water, and are lipid-soluble [133,134]. They can incorporate a variety of hydrophilic and hydrophobic drugs, enhance the accumulation of the drug at the administration site, and minimize the side effects. Therefore, liposomes have been widely used as safe and effective drug vehicles in topical applications. Liposomes have many advantages in dermal and transdermal drug delivery as they have a high solubilization capacity and a penetration enhancer effect (Figure 6). As drug delivery systems, liposomes have been used for transporting multiple therapeutical agents for vaccine immunization, cancer treatment, gene therapy, cosmetic formulations, and radiopharmaceuticals for diagnostic imaging [135,136].

Quercetin has numerous potential applications in the prevention and treatment of different diseases. The use of quercetin is limited by its low water solubility, lower bioavailability, high rate of metabolism, and rapid clearance from plasma and cells [137]. These problems can be overcome by developing a liposomal delivery system to solubilize quercetin and to enhance its pharmacological and pharmacokinetic activities as liposomes facilitate intracellular delivery and prolong the retention time of quercetin inside cells. The stability and cytotoxicity of quercetin can be enhanced by incorporating it into liposomes [138]. Quercetin-loaded liposomes possessed a good stability profile and showed a reduced tendency to adsorb water and low hygroscopicity [139].

### 4.4. Niosomes

Niosomes as microscopic lamellar structures are typically formed through the mixing of the appropriate quantities of non-ionic surfactants and calcitriol, subsequently followed by hydration in an aqueous medium [140]. The primary goal of these vesicular delivery systems is to encapsulate biologically active components to elevate their bioavailability and solubility, whereas hydrophilic compounds are encapsulated in the hydrophilic inner part. The thin-film hydration method is one of the simplest and most common ways of producing niosomal particles. Niosomes and liposomes are very similar with regard to their chemical makeup, methods of preparation, and structural characteristics. Nonetheless, niosomes offer advantages beyond just enhanced permeability and skin retention in transdermal drug delivery. They are also more stable, require less preparation, and offer sustained drug release [141].

Ionic surfactants were utilized in the production of the initial surfactant vesicles. The toxicity studies on surfactants showed that cationic surfactants are the most toxic, followed by anionic surfactants, while non-ionic surfactants are the least toxic [142]. Hence, it is important to take safety and industrial applications into consideration to ensure better transdermal delivery [143]. Niosomes with a Hydrophilic–Lipophilic Balance (HLB) magnitude range of 4 to 8 will be simpler to form because of the structure of the amphiphilic bilayers. Span 60/RH40 was used for preparing quercetin-loaded niosomes. The effectiveness of drug entrapment in quercetin-loaded niosomes has been assessed using a mini-column centrifugation technique. The antioxidant properties of the formulation have been evaluated through measurement of the DPPH bleaching rate, and it was reported that quercetin-loaded niosomes have shown good antioxidant potential [144].

### 4.5. Transferosomes

Transferosomes are a type of elastic or deformable vesicle and were first introduced in the early 1990s. The incorporation of an edge activator in the lipid bilayer structure produces elasticity. These vesicles are comprised of soya phosphatidyl choline, sodium cholate, and a small quantity of ethanol [145]. Transfersomes showed good characteristics including entrapment efficiency, zeta potential, particle size, and polydispersity index (83.0 ± 2.2%, −13.6 ± 6 mv, 75.95 ± 2 nm, and 0.333, respectively) [146].

Transfersomes are potential candidates for achieving a sustained drug release, as well as providing a predictable and extended period of activity [147]. Transferosomes overcome the main shortcomings of conventional liposomes and can penetrate pores much smaller than their own diameters. They can also increase the transdermal flux and improve the site specificity of bioactive agents. In addition, even after going through the smaller pores, the transferosomes retained their diameters without breaking apart. Their performance has improved compared to traditional liposomes because the transferosomal formulation uses EAs [148]. EAs were used to develop a transferosomal formulation in which quercetin was loaded in transferosomes in order to treat osteoporosis prior to being loaded in chitosan film. Researchers carried out ex-vivo permeation studies on Wistar rat skin, and the results of those studies showed that transferosomes improved quercetin permeation in comparison with free quercetin. Quercetin-loaded transferosomes minimize osteoclast genesis and osteoblast apoptosis, which leads to an increase in femur thickness, length, density, weight, and bone mineralization. As a result, quercetin-loaded transferosomes have been found to be a good alternative in the oral administration of quercetin to treat osteoporosis [149].

### 4.6. Nanoemulsions

Colloidal dispersion systems, known as nanoemulsions, are thermodynamically stable systems consisting of two immiscible liquids combined with emulsifying agents, such as co-surfactants and surfactants, to form a single phase. The typical range of mean droplet diameters ranges from 50 to 1000 nm. Some authors consider 500 nm to be the upper limit of the size range, while other authors do not. As a result, since there is no noticeable change in physicochemical properties when emulsion droplet size attains the nanometer range, the size limit may not be considered a significant problem. Nanoemulsions have a higher surface area and free energy that make them an effective transport system [150,151]. They do not show any complications caused by inherent creaming, flocculation, coalescence, and sedimentation. The tiny droplet size of the nanoemulsions enables them to remain stable against creaming or sedimentation. Ionic and non-ionic ethoxylated surfactants are often used in O/W nanoemulsions to stabilize against flocculation. Because of electrostatic and steric stabilization caused by their small size, nanoemulsions can penetrate through the “rough” skin surface, increasing the penetration of active ingredients. Nanoemulsions are more stable than liposomes, allowing even the development of a lamellar liquid-crystalline phase around the droplets in some cases [152]. A nanoemulsion, unlike microemulsions, is a kinetically stable emulsion. It cannot form on its own due to the fact that it is a thermodynamically unstable system; therefore, a certain amount of energy (mechanical or chemical) input is required for its formation. It can be of three types: oil-in-water (O/W), water-in-oil (W/O), and bicontinuous. By changing the emulsions’ constituent parts, it is possible to convert between these three types.

Another application that is actively being researched is the use of nanoemulsions in controlled drug delivery and targeting. The most common use of nanoemulsions is the synthesis of nanoparticles through a polymerizable monomer as the disperse phase, and nanoemulsion droplets as nanoreactors. Since quercetin is hydrophobic in nature, which makes it challenging for the body to absorb, it is one of the flavonoids that might be able to combat cancer. In order to deliver quercetin to the lungs through the pulmonary route, an oil-in-water (O/W) nanoemulsion system could prove effective [153].

Zorzi et al. designed a formulation in which they evaluated the antioxidant activity of nanoemulsions containing quercetin and extracts of *Achyrocline satureioides*. Of the flavonoids found in extracts of *Achyrocline satureioides*, the author found that quercetin exhibited the highest level of antioxidant activity [154].

A solvent displacement method was used to prepare a nanoemulsion. Quercetin’s antioxidant effect was found to have significantly increased when the formulation’s antioxidant activity was assessed using the thiobarbituric acid-reactive species (TBA-RS) assay, which uses egg yolk and AAPH (2,2-azobis(2-aminopropane) dihydrochloride). It was also discovered that when encapsulated in nanoemulsion form, just a tiny amount of quercetin in the skin was sufficient to produce an antioxidant effect.

### 4.7. Microemulsion

Microemulsions are solutions that are isotropic, macroscopically homogeneous, thermodynamically stable, and kinetically unstable. It consists of a minimum of three components: a surfactant, a nonpolar phase (typically oil), and a polar phase (generally water) [155]. Droplets in the microemulsion have a mean diameter of less than 0.22 mm. The small size of droplets in microemulsions, typically less than 100 nm, results in a very large interfacial area, from which the drug is rapidly released into the external phase when absorption (in vitro or in vivo) occurs, keeping the concentration in the external phase close to initial levels. Microemulsions serve as drug supersolvents. They can solubilize both hydrophilic and lipophilic drugs, as well as drugs that are relatively insoluble in both hydrophilic and hydrophobic solvents. The effectiveness of a drug can be enhanced by using microemulsion delivery systems, which lowers the overall dosage and minimizes side effects [156].

Due to its capacity to enhance drug solubilization, a microemulsion is a promising option for the oral administration of poorly water-soluble medications [157]. A drug’s absorption rate goes up as its thermodynamic activity in the vehicle rises. The most significant phenotypes linked to the asthma process, such as eosinophil recruitment, IL-4 and IL-5 levels in the BALF, P-selectin expression, and mucus secretion in the lung, were seen to decrease due to a quercetin-loaded microemulsion, probably as a result of its capacity to inhibit NF-B activation. Vicentini et al. designed a formulation in order to evaluate the in vivo photoprotective impacts of a w/o microemulsion containing quercetin against UV-B-induced dermal damage [158]. Quercetin has been shown to modulate NF-B activation produced by various inductors in various cell types.

**Figure 7:**
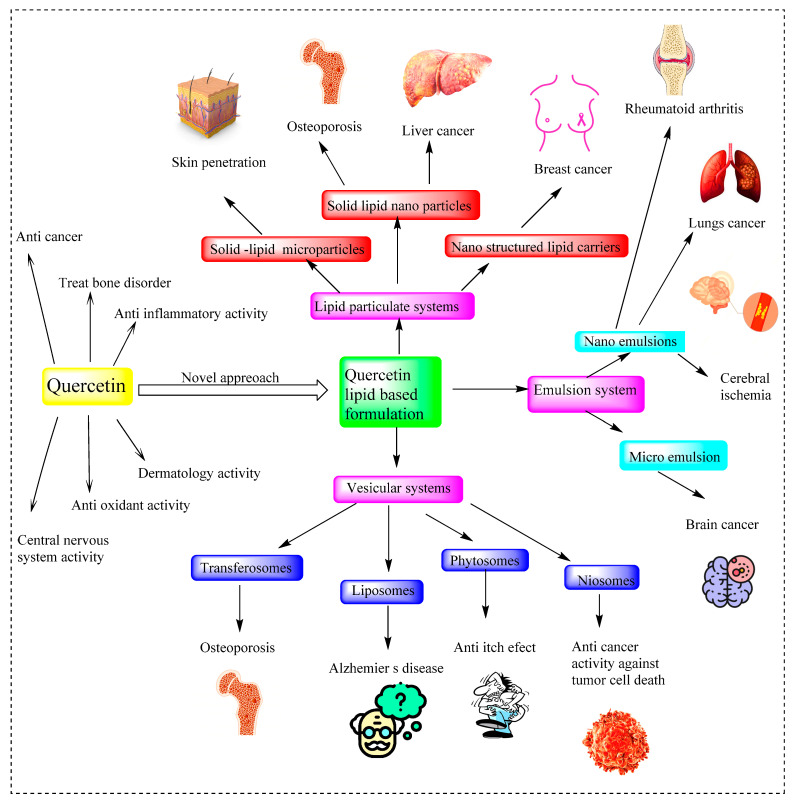
Systematic representation of multifunctional activities of Quercetin and its transition to novel lipid-based formulation for targeted delivery.

To summarize the applications of lipid nanoparticles loaded with quercetin, a diagrammatic illustration is added in the form of Figure 7, which shows the multifunctional activities of quercetin and its transition to a novel lipid-based formulation for targeted delivery.

## 5. Conclusions

Quercetin is a widely distributed flavonol compound, and it is commonly present in vegetables and fruits such as tomatoes, onions, and grapes, and green tea. Recently, quercetin has received more attention due to its potential therapeutic effects. Therefore, this review provides insights into the mechanism of action of quercetin for prostate cancer, quercetin patents, commercial products, and clinical trials, which provide a comprehensive review of the beneficial impact of quercetin as an antioxidant, anti-inflammatory, antidiabetic, anti-obesity, and protective agent in cardiovascular diseases risk. It also ameliorates immunity and shows speedy recovery from COVID-19. The literature is supportive of the therapeutic capacities of quercetin in treating prostate cancer. In conclusion, quercetin is a widespread flavonoid with a broad range of therapeutic effects on human health. The clinical trial data further advocate the narrative to explore it as a drug. So, based on the shared information, the authors are of the opinion that a full battery of safety and efficacy studies for any indication to validate the claims should be carried out. If the results are encouraging, further indications can be added to the studies, and in that case, the safety trials will be abridged.

## Figures and Tables

**Table 1:** List of biological sources of quercetin such as onion (*Allium cepa* L.), apple (*Malus domestica* (Suckow) Borkh, grapes (*Vitis vinifera* L.), etc. Quercetin occurs in different parts of these sources like bulb, peel, fruit, leaf, pomace, leaf extract, and seed.

Biological Source	Genus	Species	Authorship	Specified Parts	Concentration	Reference
Onion (*A. cepa* L.)	*Allium*	*A. cepa*	Carl Linnaeus, 1753	Bulb	79–431 mg/kg of fresh weight	[13]
Apple (*Malus* *Domestica* S.)	*Malus*	*M. domestica*	1300 BC	Pomace (peel, pulp, and seeds)	1.61 g kg^−1^ DM	[14]
Green tea (*Camellia* *Sinensis* L.)	*Camellia*	*C. sinensis*	Shen Nong, 2700 BC	Leaf extract	10–70 μg/mL	[15]
Grape (*V. vinifera* L.)	*Vitis*	*V. vinifera*	Caucasus region of Eurasia, 6000 BC	Peel extract	0.45−57.6 μg/mL	[16]
Blueberry (*Vaccinium* *corymbosum* L.)	*Vaccinium*	*V. corymbosum*	Carl Linnaeus in 1753	Fruit	59.4% ± 8.7% *w/w*	[17]
Cranberry (*Vaccinium* *Macrocarpon* Aiton.)	*Vaccinium*	*V. macrocarpon*	James Gordon in 1760	Pomace	146.2 mg/100 g of dry weight	[18]
Broccoli (*Brassica* *Oleracea* L.)	*Brassica*	*B. oleracea*	2000 BC	Whole vegetable	0.03 to 10.85 mg/100 g of fresh weight	[19]
Chinese plum (*Prunus salicina* L.)	*Prunus*	*P. salicina*	China in 470 BC	Fruit	32.7 mg/100 g of dry weight	[20]
Buckwheat (*Fagopyrum esculentum* Moench.)	*Fagopyrum*	*F. esculentum*	2600 BC	Seed	0.09–3 mg/g of fresh weight	[21]
Mulberry (*Morus alba* L.)	*Morus*	*M. alba*	220 BC	Leaf	0.452 g/100 g of dry weight.	[22]
*Moringa oleifera* L.	*Moringa.*	*M. oleifera*	Jean-Baptiste Lamarck in 1785	Leaf extract	5 to 2000 mg/mL	[23]
*Rapinus sativus* L.	*Rapinus*	*R. sativus*	Western Europe in 16th century	Leaf extract	5 to 2000 mg/mL	[24]
*Curcuma angustifolia Roxb.*	*Curcuma*	*C. angustifolia*	China in 700 BC	Leaf	4.5 µg/mg of fresh weight	[25]
*Centella asiatica* L.	*Centella*	*C. asiatica*	Southeast Asian countries	Leaf	Reported (not quantified)	[25]
*Asparagus officinalis* L.	*Asparagus*	*A. officinalis*		Root extract	150 mg/mL	[26]
*Coriandrum sativum* L.	*Coriandrum*	*C. sativum*	Carl Linnaeus in 1753	Methanolic extract	103.81 ± 4.76 mg kg^−1^	[27]
*Lactuca sativa* L.	*Lactuca*	*L. sativa*	Carl Linnaeus in 1753	Leaf	Reported (not quantified)	[28]

**Table 2:** Mechanism of action of quercetin in prostate cancer.

Mechanism of Action in Prostate Cancer	Reference
**I. Inhibition of proliferation**	[36]
Induction of G0/G1 (31.4–49.7%) and sub-G1 (19.77%) cell cycle arrest, which is caused by downregulation of cyclin D and E, CDK2, and cdc25c and upregulation of p21, p53, p18, and p27 in PC-3 and LNCaP cell lines.	
**II. Induction of apoptosis**	[38]
Increasing pro-apoptotic Bax and by decreasing anti-apoptotic Bcl-2 protein results in a significant decrease in the Bcl-2/Bax ratio.	
**III. Inhibition of the androgen receptor (AR)**	[41]
(i) Retardation of DNA synthesis and modulation of the AR signaling pathway. (ii) Reduced expression of AR and then increased caspase-3/-7 causing subsequent anti-proliferation and apoptosis in LNCaP cells.	
**IV. Inhibition of angiogenesis**	[43]
This was caused by a deleterious effect on the AKT/mTOR/P70S6K pathway. Quercetin decreases angiogenesis in LNCaP cells by lowering HIF-1α accumulation and VEGF release.	

## Data Availability

Not applicable.

## Supplementary Materials

The following supporting information can be downloaded at: https://www.mdpi.com/article/10.3390/ph16111631/s1, Table S1: Globally available marketed products of quercetin (products ranges from 100 mg to 1500 mg). Table S2. Depicts commercial products of quercetin.
