# Supplementary material for: Quercetin as a Therapeutic Product: Evaluation of Its Pharmacological Action and Clinical Applications—A Review
Source: Pharmaceuticals (Basel). 2023 Nov 20;16(11):1631. doi: 10.3390/ph16111631 (PMC10674654; doi:10.3390/ph16111631)
Supplement: Supplementary file 1 [file pharmaceuticals-16-01631-s001.zip › pharmaceuticals-2658914-supplementary.pdf]

Supporting Information

Table S1. Globally available marketed products of quercetin (products ranges from 100 mg to 1500 mg).

S.No	Product	Serving Size (and form)	Amount per serving	Net Contents	Website
1.	Absorb Health Quercetin	1.0 Capsule(s)	500 mg	100 Capsule(s)	www.absorbyourhealth.com
2.	Allergy Research Group Quercetin	2.0 Capsule(s)	300 mg	60 Vegetarian Capsule(s)	www.allergyresearchgroup.com
3.	Allergy Research Group Quercetin Bioflavonoids	2.0 Capsule(s)	100 mg	100 Vegetarian Capsule(s)	www.allergyresearchgroup.com
4.	AN Amazing Nutrition Amazing Formulas Quercetin	1.0 Veggie Capsule(s)	1,500 mg	120 Veggie Capsule(s)	www.amazingnutrition.com
5.	Bio-Tech Pharmacal Quercetin	1.0 Capsule(s)	400 mg	100 Vegetable Capsule(s)	www.BioTechPharmacal.com
6.	BIOVEA Quercetin	2.0 Vegetarian Capsule(s)	500 mg	50 Vegetarian Capsule(s)	www.biovea.com
7.	Bluebonnet Super Quercetin	2.0 Capsule(s)	500 mg	30 Vegetable Capsule(s)	www.bluebonnetnutrition.com
8.	Body Kitchen Mega NAC + Quercetin	2.0 Vegetable Capsule(s)	600 mg	60 Capsule(s)	www.mybodykitchen.com
9.	Bronson Quercetin with Bromelain	2.0 Capsule(s)	500 mg	100 Capsule(s)	www.BronsonVitamins.com
10.	Bronson Laboratories Quercetin & Bromelain	1.0 Capsule(s)	250 mg	100 Capsule(s)	
11.	Bulk Supplements	500.0 mg [about 1/2 tsp]	500 mg	1.0 KG(s); 35.3 Ounce(s)	https://www.bulksupplements.com
12.	Country Life Quercetin + D3	1.0 Capsule(s)	500 mg	90 Vegetarian Capsule(s)	www.countryLifeVitamins.com
13.	Designs for Health Quercetin + Nettles	3.0 Capsule(s)	600 mg	90 Vegetarian Capsule(s)	www.designsforhealth.com
14.	Designs for Health Quercetin- Ascorbate Powder	1.0 Gram(s) [approx. 1/4 teaspoon]	500 mg	100 Gram(s)	www.designsforhealth.com
15.	Doctor's Best Quercetin Bromelain	2.0 Veggie Capsule(s)	500 mg	180 Veggie Cap(s)	www.drbvitamins.com
16.	Double Wood Supplements Quercetin with Bromelain	2.0 Capsule(s)	1000 mg	120 Capsule(s)	www.doublewoodsupplements.com
17.	Douglas Laboratories Quercetin Bromelain Complex	1.0 Tablet(s)	333 mg	100 Tablet(s)	www.douglaslabs.com
18.	Dr. Mercola Quercetin and Pterostilbene Advanced	2.0 Capsule(s)	Quercetin Phytosome Complex from Sophora japonica, contains 34.0% Quercetin	180 Capsule(s)	
19.	Forest Leaf Quercetin Bromelain	2.0 Capsule(s)	500 mg	90 Vegetable Capsule(s)	www.ForestLeaf.com

20.	Forest Leaf Quercetin Bromelain	2.0 Capsule(s)	Quercetin, Phospholipid Lecithin 250 mg	90 Vegetable Capsule(s)	www.globalhealing.com
21.	GNC Preventive Nutrition Quercetin	1.0 Caplet(s)	1000 mg	60 Caplet(s)	www.GNC.COM
22.	Herbadiet Quercetin	1.0 Capsule(s)	400 mg	60 V-Cap(s)	www.herbadiet.in
23.	Herbal Secrets Quercetin	1.0 V-cap(s)	500 mg	120 V-cap(s)	www.herbalsecrets.com
24.	Innate Response Formulas Quercetin	1.0 Tablet(s)	500 mg	90 Tablet(s)	www.innateresponse.com
25.	Jarrow Formulas Quercetin	1.0 Capsule(s)	500 mg	200 Veggie Cap(s)	www.jarrow.com
26.	KAL Quercetin	1.0 Tablet(s)	1000 mg	60 Tablet(s)	www.nutrafamily.com
27.	Mega Food Quercetin Strength	1.0 Tablet(s)	500 mg	30 Tablet(s)	www.megafood.com
28.	Micro Ingredients Pure Quercetin Dihydrate	0.5 Gram(s) [1 scoop]	500 mg	100 Gram(s); 3.53 Oz(s)	www.microingredients.com
29.	Moss Nutrition Quercetin	1.0 Capsule(s)	300 mg	60 Vegetarian Capsule(s)	www.mossnutrition.com

Table S2. Depicts commercial products of quercetin.

S.No.	Product	Net content	Recommended daily Dose	Amount of Quercetin/ Serving Unit	Manufacturer/ Distributor	Website
1.	Quercetin with Bromelain	60 vegetarian capsules	As a Dietary Supplement for adults, 1 capsule before meal, 2 times daily or as directed by health practitioner.	400 mg	Biotrex Nutraceuticals, India	https://www.amazon.in/s?k=quercetin+marketed+products&ref=nb_sb_noss
2.	"Diagnose to Cure" Quercetin Plus	60 vegetarian capsules	1-2 Capsules with water after meal.	500 mg	Diagnose to Cure, India	https://www.amazon.in/s?k=quercetin+marketed+products&ref=nb_sb_noss
3.	Healthvit Quercetin, Natural Bioflavonoid	60 capsules	As a dietary supplement, take 2 capsules 20 minutes before meals,2-3 times daily. person with sensitive stomach may take capsule with meals. Warning: If you are pregnant, nursing or taking any medication, consult your doctor before use.	100 mg	West Coast Pharmaceutical Works Ltd, India	https://www.amazon.in/s?k=quercetin+marketed+products&ref=nb_sb_noss
4.	Quercetin With Bromelain	60 vegetarian Capsules	1 veg capsule Daily value (DV) not established	500 mg	Back To Beginnings, India	https://www.amazon.in/s?k=quercetin+marketed+products&ref=nb_sb_noss

5.	Citokain, Quercetin and Bromelain	60 capsules	Dose:3 times a day daily. For adult:1 capsule after breakfast,1 capsule after lunch ,1 capsule after dinner. For children- age 5-10 yr - 1 capsule/ day Age-10 to 15 yrs 2capsule/day	Quercetin-350mg Bromelain -350mg	Dr. Pravin Rane, India (Maharashtra)	https://www.amazon.in/s?k=quercetin+marketed+products&ref=nb_sb_noss
6.	Now Foods, Quercetin with Bromelain	240 veg capsules	Take 2 capsules 20 min before meals, twice daily. Person with sensitive stomach may take capsule with meals. For adults only	Quercetin 800 mg * Bromelain (2400 GDU/g) 165 mg	Now Foods, USA	https://www.amazon.in/s?k=quercetin+marketed+products+in+usa&ref=nb_sb_noss
7.	Quercetin Complex	100 vegetable capsules	As a dietary supplement for adults, take two (2) vegetable capsules twice daily, preferably with a meal or as directed by a healthcare practitioner. Not intended for use by pregnant or lactating women.	500 mg	Solgar, USA	https://www.amazon.in/s?k=quercetin+marketed+products+in+usa&ref=nb_sb_noss
8.	Jarrow Formulas, Quercetin	100 veggie capsules	take 1 capsule per day with meals or as directed by your qualified healthcare professional	500 mg	Jarrow Formulas, USA	https://www.amazon.in/s?k=quercetin+marketed+products+in+usa&ref=nb_sb_noss
9.	Natrol - Quercetin	50 capsules, Non-vegetarian product	Take 2 capsule, one time daily with a meal. Not intended for use by pregnant or lactating women	500mg Daily value not established	Natrol, USA	https://www.amazon.in/s?k=quercetin+marketed+products+in+usa&ref=nb_sb_noss
10.	Quercetin Brand: Kal	60 tablets	Take 1 tablet with a meal or a glass of water. Store in cool and dry place. Use only as Directed	1000 mg (Daily value not established)	Nutraceutical corp. Kal, USA	https://www.amazon.in/s?k=quercetin+marketed+products+in+usa&ref=nb_sb_noss
11.	Source Naturals: Activated Quercetin	100 tablets	3 tablets. 1,2 times daily in divided doses. if you have a delicate, easily irritated	1 g	Source Naturals, USA	https://www.amazon.in/s?k=quercetin+marketed+products+in+usa&ref=nb_sb_noss
12.	Doctor's Best, Quercetin Bromelain	180 veggie capsules	Take 2 capsules daily, preferably between meals. For added benefit take 2 capsules up to 3 times daily	500mg Daily value not established	Doctor's Best, California, USA	https://www.amazon.in/s?k=quercetin+marketed+products+in+usa&ref=nb_sb_noss

13.	Quercetin with Bromelain<br><br>Brand: Now Foods (Dietary supplement)	120 veggie capsules	Take 2 capsules 20min before meals, twice daily. Person with sensitive stomach may prefer to take capsule with Food	800mg	Now Foods, USA	https://www.amazon.in/s?k=quercetin+marketed+products+in+usa&ref=nb_sb_noss
14.	Solaray Quercetin Capsules	90 veg capsules	1 veg capsule. Daily value not established	500mg	Solaray, New Jersey, USA	https://www.amazon.in/s?k=quercetin+marketed+products+in+usa&ref=nb_sb_noss
